# Gel-Based PVA/SiO_2_/p-Si Heterojunction for Electronic Device Applications

**DOI:** 10.3390/gels10080537

**Published:** 2024-08-20

**Authors:** Adel Ashery, Ahmed E. H. Gaballah, Gamal M. Turky, Mohamed A. Basyooni-Murat Kabatas

**Affiliations:** 1 Solid State Physics Department, Physics Research Institute, National Research Centre, 33 El-Bohouth St, Dokki, Giza 12622, Egypt; 2 Photometry and Radiometry Division, National Institute of Standards (NIS), Tersa St, Al-Haram, Giza 12211, Egypt; 3 Microwave Physics and Dielectrics Department, Physics Research Institute, National Research Centre, Behooth St, Dokki, Giza 12622, Egypt; 4 Department of Precision and Microsystems Engineering, Delft University of Technology, Mekelweg 2, 2628 CD Delft, The Netherlands; 5 Department of Nanotechnology and Advanced Materials, Graduate School of Applied and Natural Science, Selçuk University, Konya 42030, Turkey; 6 Solar Research Laboratory, Solar and Space Research Department, National Research Institute of Astronomy and Geophysics, Cairo 11728, Egypt

**Keywords:** gel materials, heterojunction diode, I–V and C–V characterization, polyvinyl alcohol (PVA), polymer oxide semiconductor

## Abstract

The current work presents a new structure based on Au/PVA/SiO_2_/p-Si/Al that has not been studied before. An aqueous solution of polyvinyl alcohol (PVA) polymer gel was deposited on the surface of SiO_2_/Si using the spin-coating technique. The silicon wafer was left to be oxidized in a furnace at 1170 k for thirty minutes, creating an interdiffusion layer of SiO_2_. The variations in the dielectric constant (Є′), dielectric loss (Є″), and dielectric tangent (tanδ) with the change in the frequency, voltage, and temperature were analyzed. The results showed an increase in the dielectric constant (Є′) and a decrease in the dielectric loss (Є″) and tangent (tanδ); thus, the Au/PVA/SiO_2_/p-Si/Al heterostructure has opened up new frontiers for the semiconductor industry, especially for capacitor manufacturing. The Cole–Cole diagrams of the Є″ and Є′ have been investigated at different temperatures and voltages. The ideality factor (n), barrier height (Φ_b_), series resistance (R_s_), shunt resistance (R_sh_), and rectification ratio (RR) were also measured at different temperatures.

## 1. Introduction

In recent years, there has been significant progress in developing materials and techniques for sensor applications, particularly those involving thin films and nanostructures [1,2]. Polymer oxide semiconductor (POS) structures consist of a thin oxide film between a polymer and a semiconductor [3]. The oxide layer prevents inter-diffusion between the polymer and semiconductor substrate and improves the electric field reduction inside the structures [4,5,6]. It enhances the electrical and dielectric properties of devices such as capacitors [7,8,9], which increases the electric charge’s storage capacity. The creation of oxide thin films on Si substrates using traditional methods of oxidation or deposition cannot passivate the active hanging bonds on the surface of semiconductors [10,11]. The carrier lifetime remains more prolonged at high angular frequencies than the period (T = 1/x). Thus, the charges at the border states cannot track an AC sign [12]. Contrarily, the charges at minor frequencies could follow the AC signal, and thus, the effect of these charges on the capacitance of devices increases with decreasing frequency [13].

Consequently, the electrical and dielectric characteristics depend on the frequency, making accuracy and trust results very significant. The interface states at the Si/SiO_2_, PVA/SiO_2_ interfaces, and device contacts cause deviation from the perfect case [14,15]. These interfaces commonly cause a bias change and frequency dispersal of the C–V and G–V curves [16]. Thus, it is noteworthy to incorporate the effect of the frequency and thoroughly inspect the frequency dispersal of the dielectric properties. The frequency dependence of the dielectric constant (Є′), dielectric loss (Є″), and dielectric tangent (tanδ) is dominated by a small frequency dispersal, whose physical origin has long been in question. The innovation in this paper is that we have synthesized the Au/PVA/SiO_2_/p-Si/Al structure, which has not been investigated before. We present a whole study of the dielectric constant (Є′), dielectric loss (Є″), and dielectric tangent (tanδ) with the variation of the frequency, voltage, and temperature. We also succeeded in improving the dielectric constant (Є′) and reducing the dielectric loss (Є″) and the tangential component (tanδ); thus, the current structure is considered a promising material for the development of capacitors. The Cole–Cole diagrams of the Є″ and Є′ at different voltages and temperatures have been examined. In addition to that, the I–V measurements were performed to retrieve information about its electrical properties, such as the ideality factor (n), barrier height (Φ_b_), series resistance (R_s_), shunt resistance (R_sh_), and rectification ratio (RR) over different temperatures.

## 2. Results and Discussion

### 2.1. Scanning Electron Microscope and X-ray Diffraction Pattern

**Figure 1:**
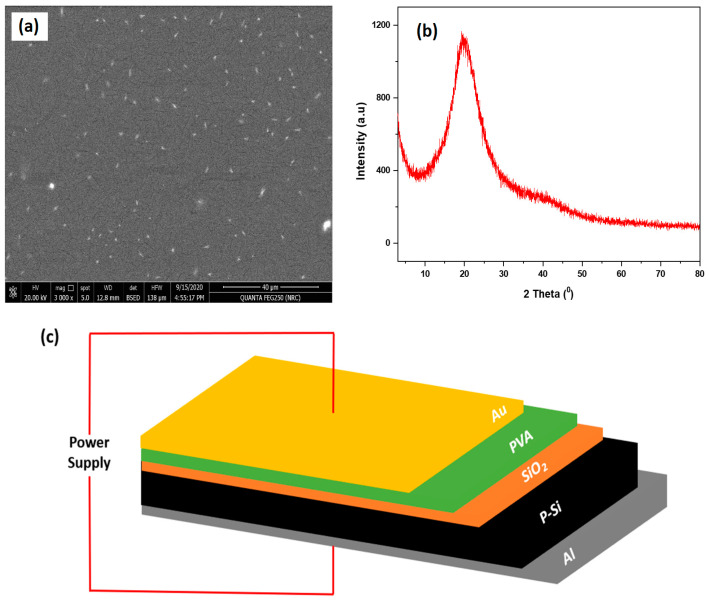
(**a**) SEM of the Au/PVA/SiO_2_/p-Si/Al structure, (**b**) XRD of the Au/PVA/SiO_2_/p-Si/Al structure, and (**c**) Au/PVA/SiO_2_/p-Si/Al structure.

SEM Analysis: Figure 1 shows the surface topography of the PVA layer on the SiO_2_/Si substrate. The SEM micrograph reveals a uniform distribution across the surface, with some cracks visible. These cracks are assumed to have originated from the drying process of the PVA film as the solvent evaporated, causing shrinkage and resulting in stress-induced cracks. The surface morphology of the PVA is relatively smooth, with minimal porosity, indicating a dense film formation.

**Figure 2:**
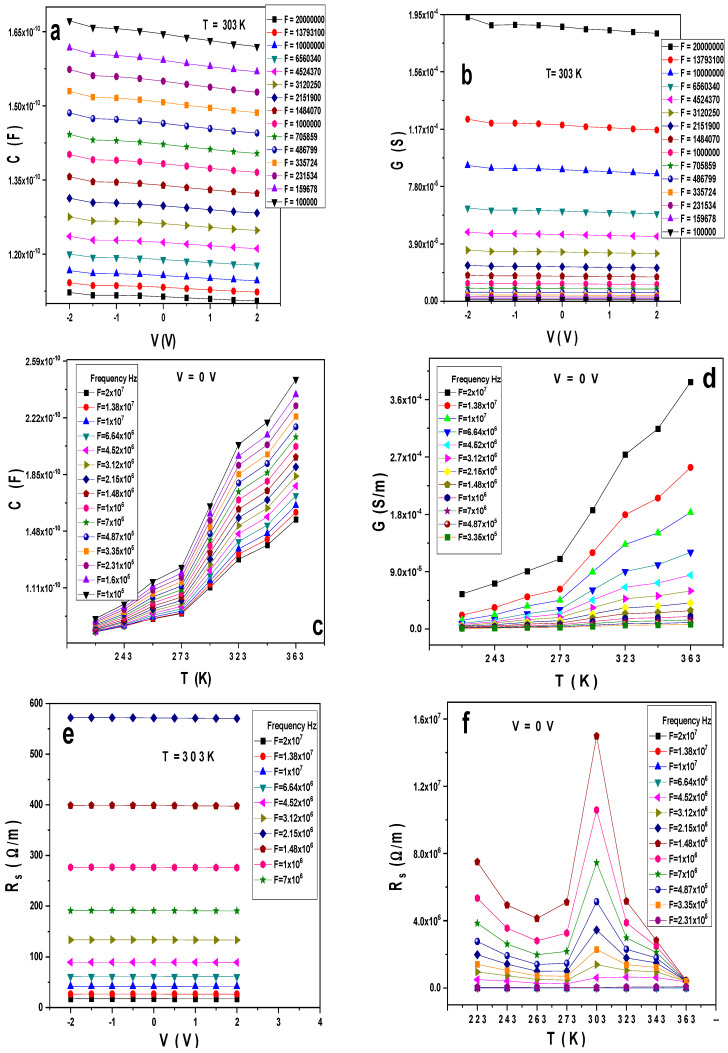
(**a**–**f**) The experimental (**a**) C–V, (**b**) G–V, (**c**) C–T, (**d**) G–T, (**e**) Rs–V, and (**f**) Rs–T for the Au/PVA/SiO_2_/p-Si/Al structure.

XRD Analysis: Figure 2 presents the XRD pattern of the PVA/SiO_2_/Si thin film. The XRD spectrum exhibits a prominent peak at 2θ = 19.7°, which is characteristic of the semi-crystalline nature of the PVA film. The broadening of the halo and the absence of sharp diffraction peaks confirm the semi-crystalline structure of the PVA, suggesting that the material has both amorphous and crystalline regions [17].

Additionally, the I–V and C–V characteristics were measured using a Keysight model B2901A and a Novocontrol high-resolution alpha analyzer. These measurements were supported by a Quatro temperature controller, which maintained the temperature stability to within 0.2 K using clean nitrogen as a heating agent.

### 2.2. Dielectric Characterization

Figure 2a,b display the variation of the capacitance (C) and conductance (G) with the voltages and frequencies at a temperature of 303 K for the Au/PVA/SiO_2_/p-Si/Al structure. As observed in Figure 2a, the capacitance decreases with the frequency, while the conductance increases. C and G are still independent of the voltage changes. At the same time, the conductance might be attributed to the spreading profile of the interface density of states at the PVA/p-Si and PVA/SiO_2_ interface and series resistance (Rs) [18,19]. Figure 2c,d display the variation of C and G with the temperature at different frequencies and a constant voltage V = 0 V; both rise with the temperature. The variation of the series resistance (R_s_) with the voltage at different frequencies at room temperature is seen in Figure 2e; it decreases with the frequencies, while Rs shows peaks at room temperature, as displayed in Figure 2f, and then decreases at high temperatures.

**Figure 3:**
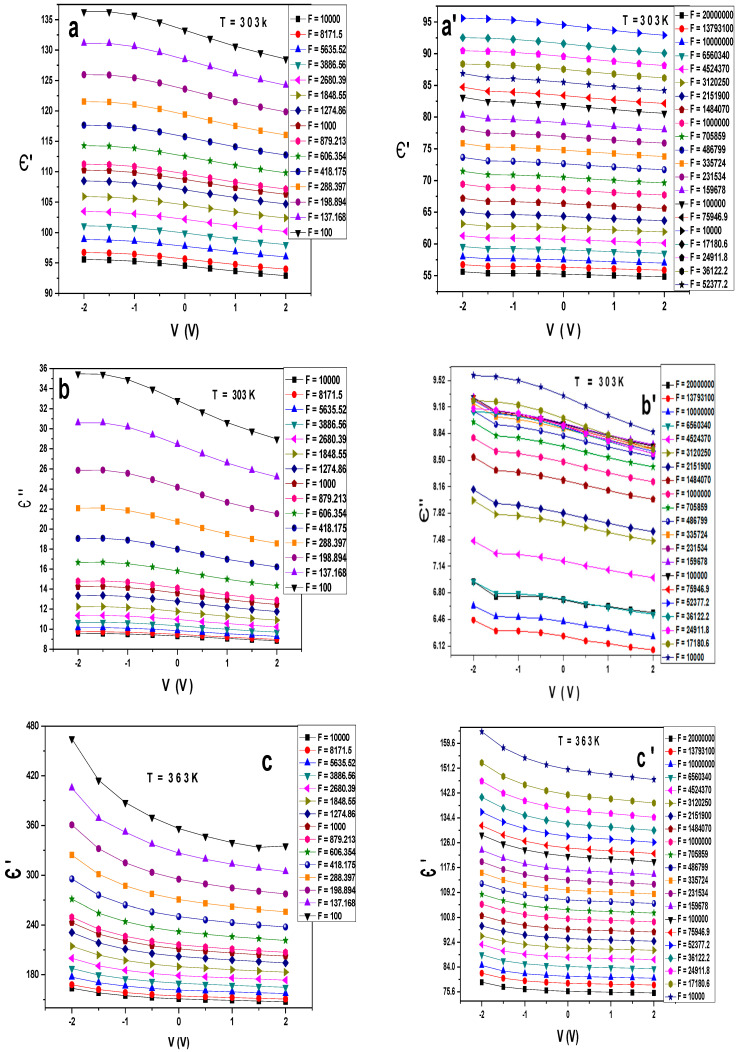

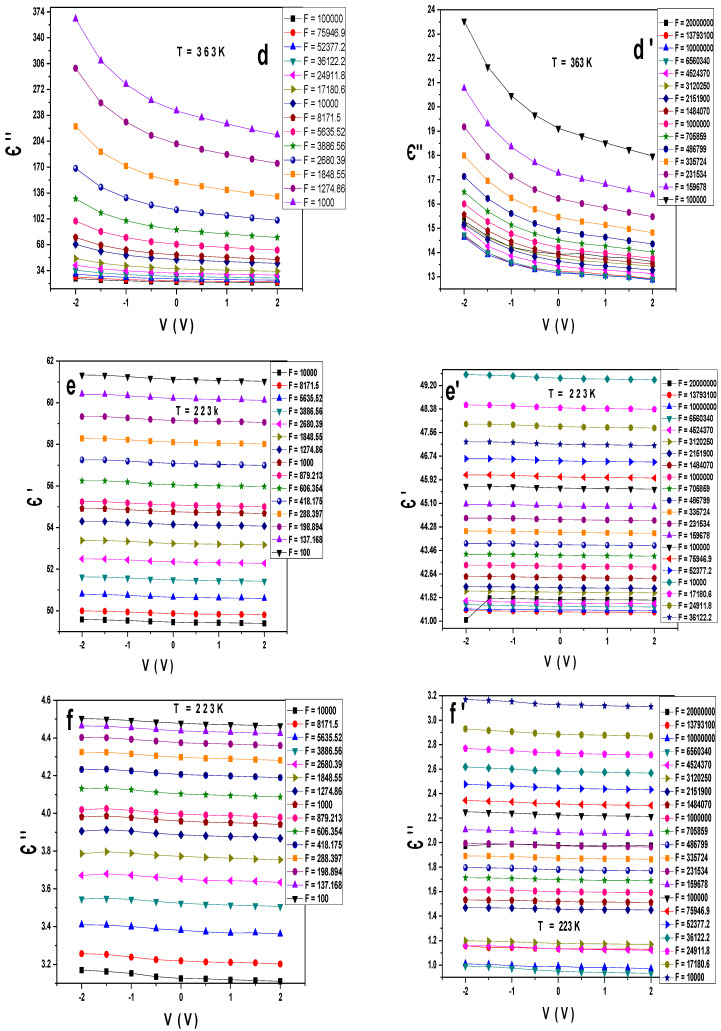
(**a**–**f**’) Є′, Є″ versus V at different frequencies and different constant temperatures for the Au/PVA/SiO_2_/p-Si/Al structure.

**Figure 4:**
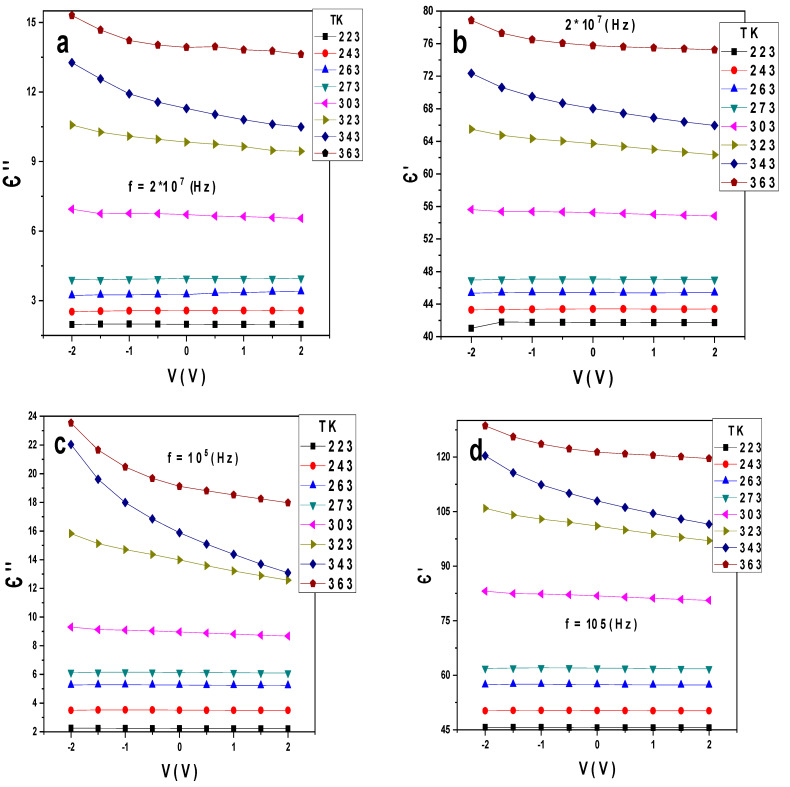

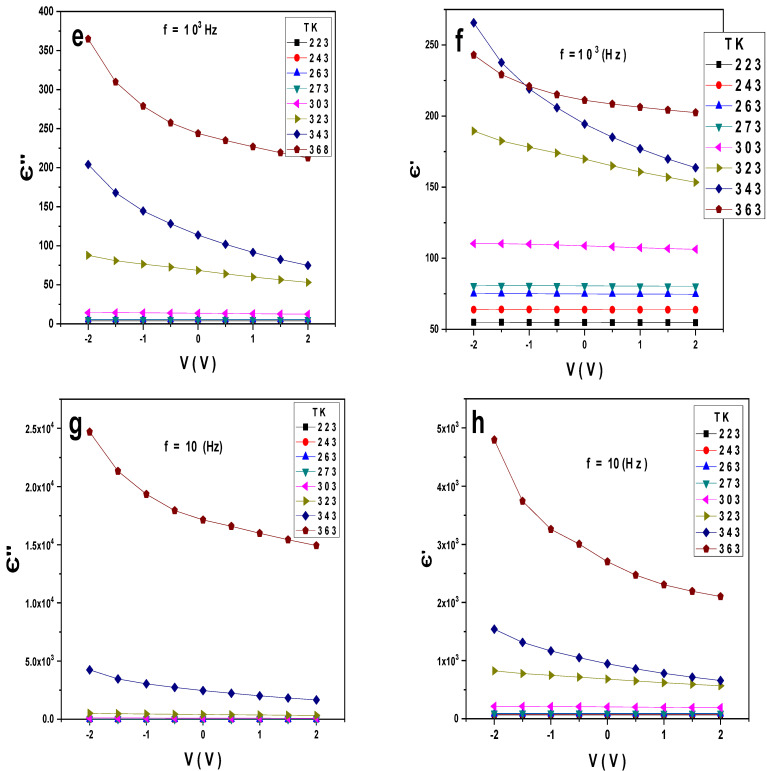
(**a**–**h**) Є′, Є″ versus V at different temperatures and constant frequencies for the Au/PVA/SiO_2_/p-Si/Al structure.

Figure 3a–b’ display the variation of the dielectric constant Є′ and Є″ at different frequencies and room temperature for Au/PVA/SiO_2_/p-Si/Al. It is seen that Є′ decreases with the frequencies, and its value ranges from 56 to 135, while the dielectric loss Є″ decreases with the frequency, taking values from 6 to 36. Here, we enhanced the values of Є′ and reduced the values of Є″, as shown in Figure 3b,b’. At a temperature of 363 K, Є′ increases from 75 to 480 while Є″ reduces from 13 to 380, as illustrated in Figure 4c,d. On the other hand, working at a low temperature equal to 223 k, the values of Є′ and Є″ range from 41 to 60 and from 1 to 4.4, respectively, as illustrated in Figure 3e–f’. As described above, we can increase the dielectric constant’s value and reduce the dielectric loss’s value with varying temperatures and frequencies. Still, their values are independent of the change in the voltage. It is stated that the values of Є′ and Є″ depend on several factors related to the interface state density, interfacial layer thickness, series resistance, doping concentration, etc. [17,20].

Figure 4a–h illustrate the variation of Є′ and Є″ with the voltages at different temperatures and constant different frequencies for Au/PVA/SiO_2_/p-Si/Al. At the frequency 2 × 10^7^ Hz, Є′ and Є″ increase with the temperatures and their values range from 40 to 80 and 3 to 15, respectively. The dielectric constant and dielectric loss dependence on the voltage is almost low, as shown in Figure 4a,b. At the frequency 10^5^ Hz, the values of Є′ and Є″ range from 45 to 120 and 2 to 24, respectively, as displayed in Figure 4c,d, while at the frequencies 10^3^ and 10 Hz, Є″ increases to large undesirable values, as shown in Figure 4e–h. These variations in the conductance of Є′ and Є″ can also be due to polarization mechanisms such as Maxwell–Wagner [21] and space charge [22,23]. Through the above, it can be concluded that at high frequencies, the value of the dielectric loss is minimal. The industry needs to reduce the value of the heat loss at medium and low frequencies as the value of constant loss rises, which is undesirable.

**Figure 5:**
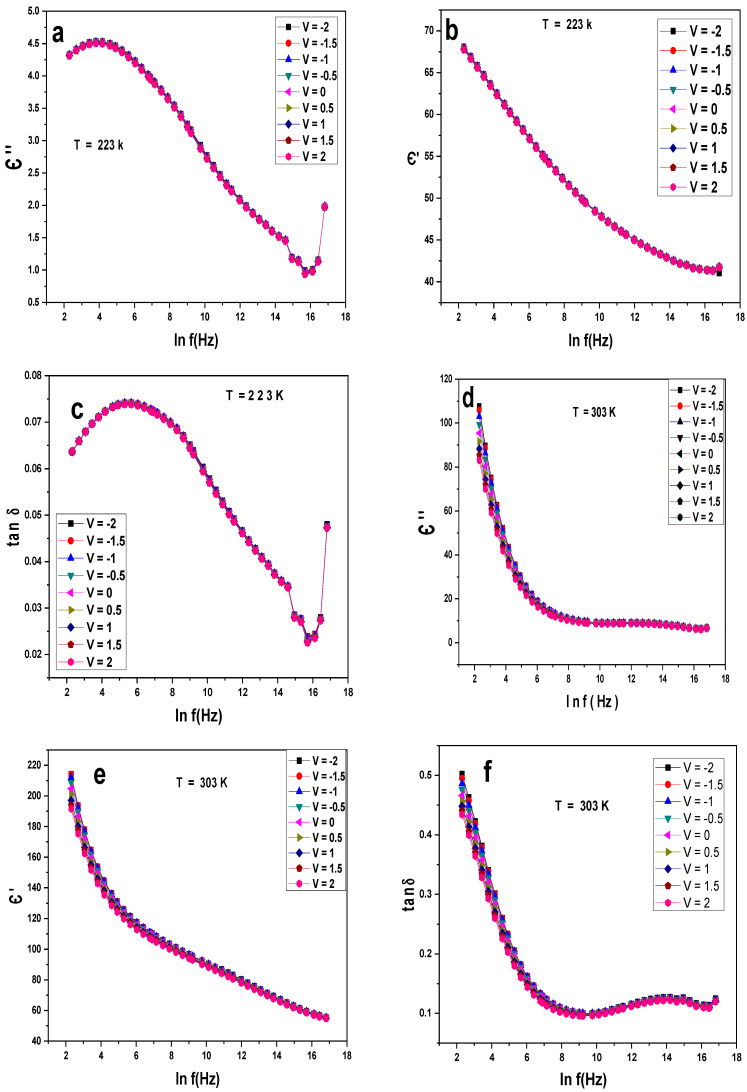
(**a**–**f**) Є′, Є″, tanδ versus lnf at different voltages and constant temperatures for the Au/PVA/SiO_2_/p-Si/Al structure.

Figure 5a–f illustrate the variation of Є′, Є″, and tanδ with the frequency at different voltages and fixed temperatures for Au/PVA/SiO_2_/p-Si/Al. At a low temperature of 223 k, the behavior is the same for all the Є′, Є″, and tanδ, and the difference is that Є″ and tanδ have two peaks at low and high frequencies. The most significant finding here was the enhancement of the value of Є′ in the range of 40–70 and the decrease in the values of Є″ in the range of 0.5–5 and 0.02–0.08, respectively, as seen in Figure 5a–c. At room temperature, Є″ and tanδ exhibit the same standard behavior, while Є′ decreases with all the frequencies. However, the values of Є′ increase compared to Figure 5a, while Є″ and tanδ decrease, as shown in Figure 5d–f. The robust reduction in Є′ and Є″ with the frequency can be described by the Debye relaxation in terms of the polarity alignment and interface effect [8]. Though a great Є is significant for high-capacity energy storage dielectrics, the balance between a high dielectric constant and a low dielectric loss is more critical [24].

**Figure 6:**
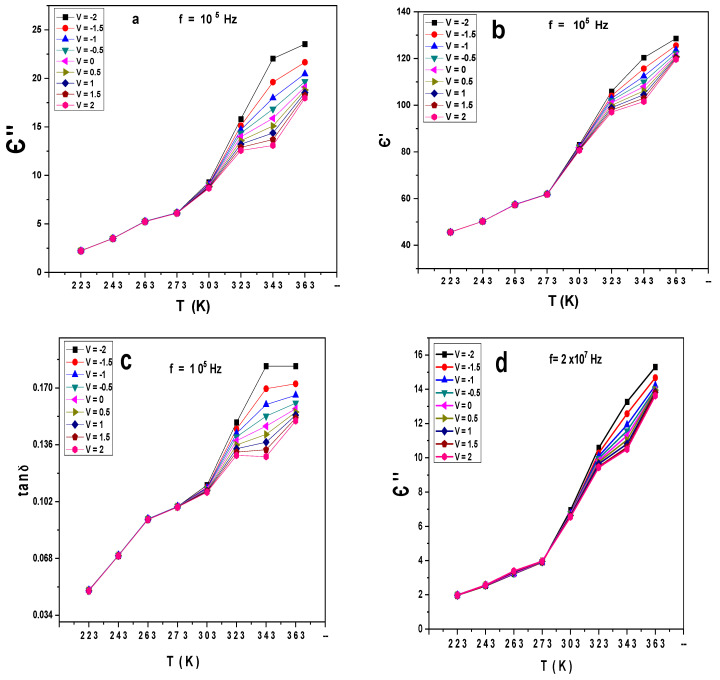

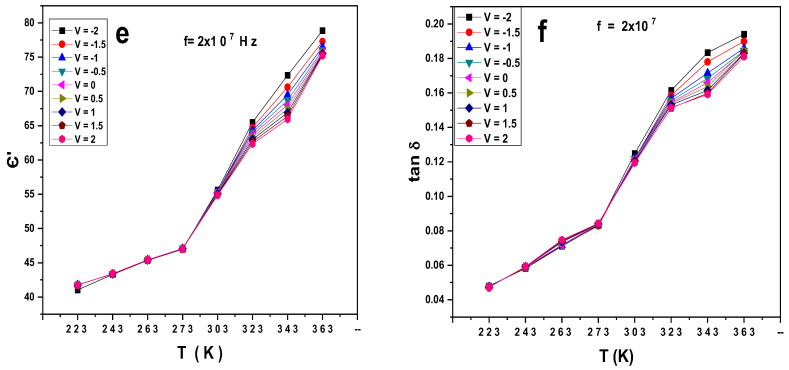
(**a**–**f**) Є′, Є″, tanδ versus Tk at different voltages and constant different frequencies (10^5^, 2 × 10^7^ Hz, respectively) for the Au/PVA/SiO_2_/p-Si/Al structure.

Figure 6a–f illustrate the variation of Є′, Є″, and tanδ with the temperature at different voltages and frequencies for the Au/PVA/SiO_2_/p-Si/Al structure. Є′, Є″, and tanδ increase with the temperature. All the curves overlap at low temperatures, while at room and high temperatures, the Є′, Є″, and tanδ curves split at each voltage. At the frequency 10^5^ Hz, the values of Є′ increase from 40 to 140, while Є″ and tanδ reduce from 0 to 25 and 0.04 to 0.2, respectively, as displayed in Figure 6a–c. As the frequency increases to 2 × 10^7^ Hz, the values of Є′, Є″ and tanδ range from 40 to 80, 0 to 16, and 0.04 to 0.2, respectively, as shown in Figure 6e,f. The impurities, disorders, or additional phases might be clarified in the construction. With an increase in temperature, the joint effect leads to a rise in the values of Є′ and Є″. This may be attributed to the ion jump and space charge effects induced by rising concentrations of the charge carriers. Also, the rising temperature enhances the growth of molecules. It can be supposed that this structure enhanced the dielectric properties Є′, Є″, and tanδ with annealing [25,26]. It is clear that with increasing frequency, Є′ has shown significant improvements, while Є″ and tanδ are decreased, increasing capacitors’ ability to store energy and reduce heating loss.

**Figure 7:**
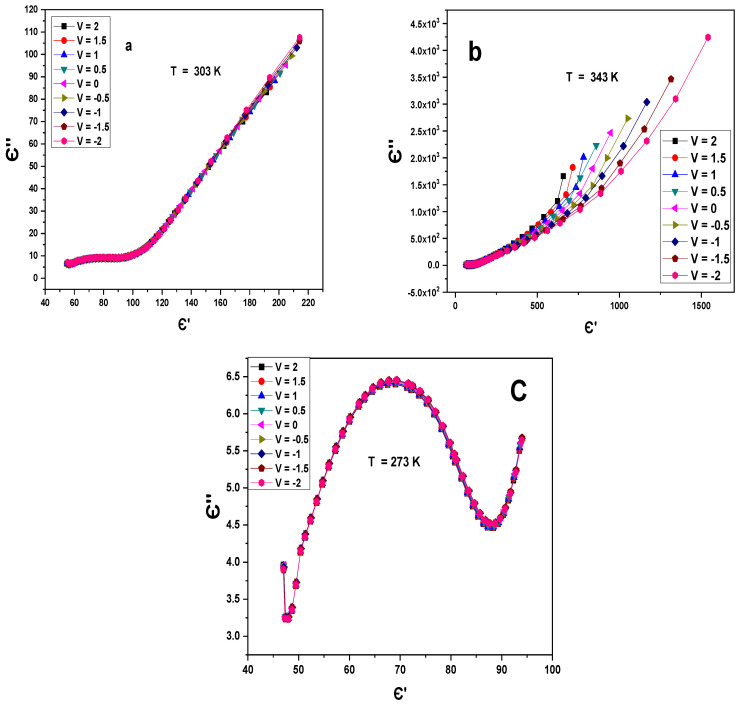
(**a**–**c**) Є″-Є′ at different voltages and constant temperatures for the Au/PVA/SiO_2_/p-Si/Al structure.

Figure 7 displays the variation of the dielectric loss (Є″) with a dielectric constant (Є′) of impedance at different voltages and constant temperatures for Au/PVA/SiO_2_/p-Si/Al. Entirely, the figures display a reduction in the radius of the partly shaped semicircles and shrinkage near the origin due to the rising ionic conductivity and declining resistance of the films [27,28].

### 2.3. The Current–Voltage (I–V) Characteristic

The current–voltage characteristics of the diode were explained according to the thermionic emission model [29,30,31]:

(1) $$I=I_{0}\left[\exp\;\frac{q\left(V-{\mathrm{IR}}_{s}\right)}{\mathrm{nkT}}\right]$$

**Figure 8:**
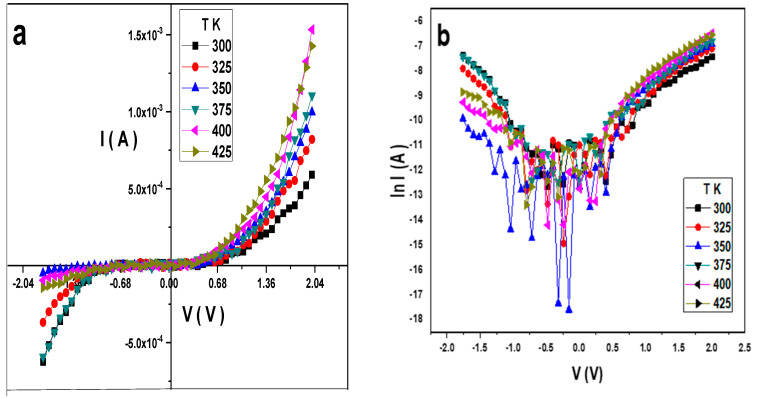
(**a**) I–V, and (**b**) lnI–V at different temperatures for the Au/PVA/SiO_2_/p-Si/Al structure.

Figure 8a illustrates the I–V characteristic for the Au/PVA/SiO_2_/p-Si/Al heterostructure diode at different temperatures, while Figure 8b displays the diode’s lnI–V semilogarithmic behavior.

The barrier height can be expressed by [32,33]:

(2) $$\varphi_{b}=\frac{\mathrm{kT}}{q}\mathrm{Ln}\;\left(\frac{{\mathrm{AA}}^{*}T^{2}}{I_{0}}\right)$$

where I_0_ is the saturation current, V is the applied voltage, R_s_ is the series resistance, n is the ideality factor, T is the temperature in Kelvin, q is the electronic charge, k is the Boltzmann constant, φ_b_ is the Schottky barrier height, A* is the Richardson constant, and A is the contact area of the diode. The intersection of the inserted straight lines of the linear part with the current axis obtained the I_0_. The ideality factor (n) can be determined using Equation (1) as [33,34]:

(3) $$n=\frac{q}{\mathrm{KT}}\left(\frac{\mathrm{dV}}{\mathrm{dlnI}}\right)$$

**Figure 9:**
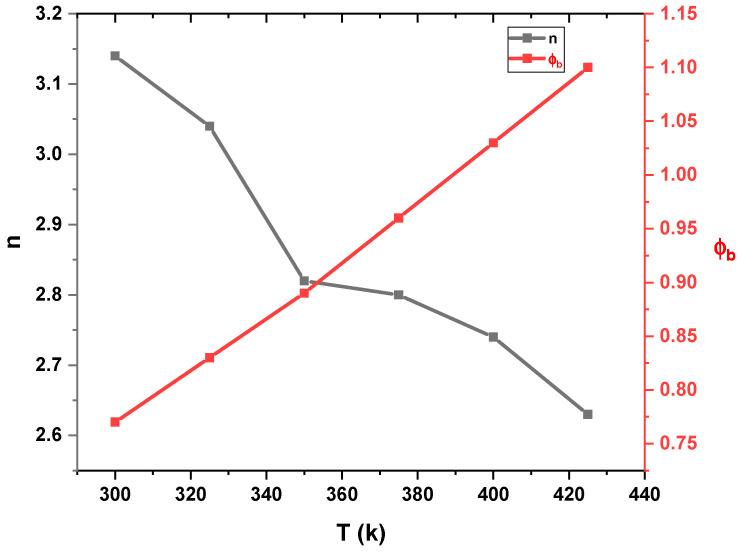
n and ɸ_b_ versus T for the Au/PVA/SiO_2_/p-Si/Al structure.

**Figure 10:**
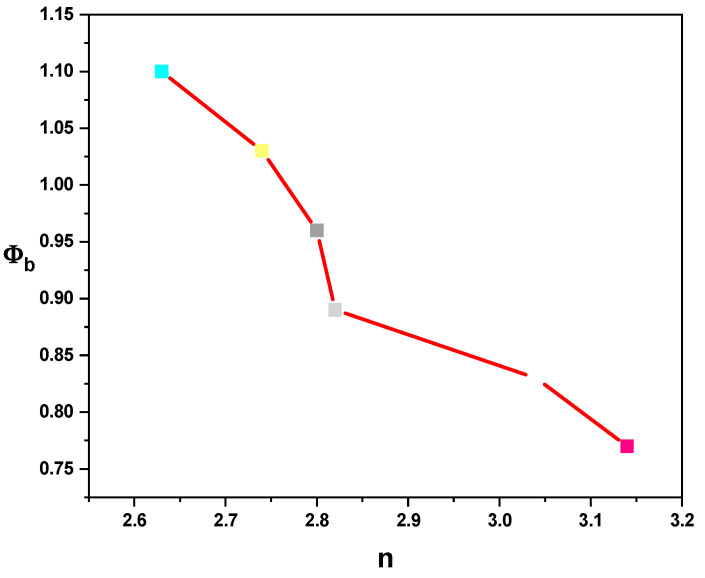
ɸ_b_ versus n for the Au/PVA/SiO_2_/p-Si/Al structure.

From Equations (1) and (2), ɸ_b_ increases with the temperature, while n decreases, as illustrated in Figure 9, and their values are listed in Table 1. Based on Equation (3), the high value of the ideality factor was attributed to the presence of a thin oxide layer and series resistance [23]. Since the non-homogeneous barrier height contributes to the higher value of the ideality factor [35,36], the higher value of n can be explained in terms of secondary mechanisms such as border dips and interfacial imperfections, which are produced by an organic interlayer or a specific border structure [37]. The variation of the barrier height with the ideality factor is illustrated in Figure 10.

**Figure 11:**
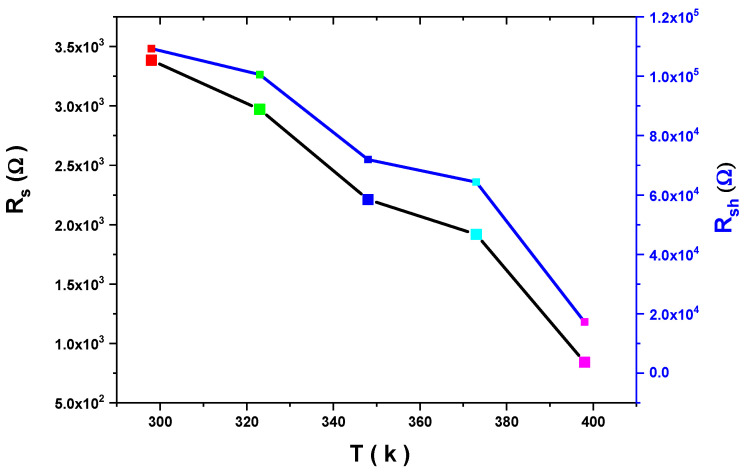
R_s_ and R_sh_ versus T for the Au/PVA/SiO_2_/p-Si/Al structure.

**Figure 12:**
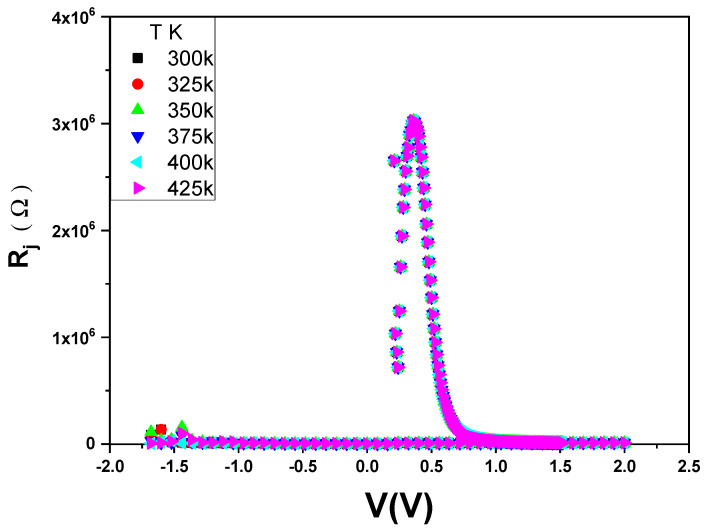
R_j_ versus V at different temperatures for the Au/PVA/SiO_2_/p-Si/Al structure.

**Figure 13:**
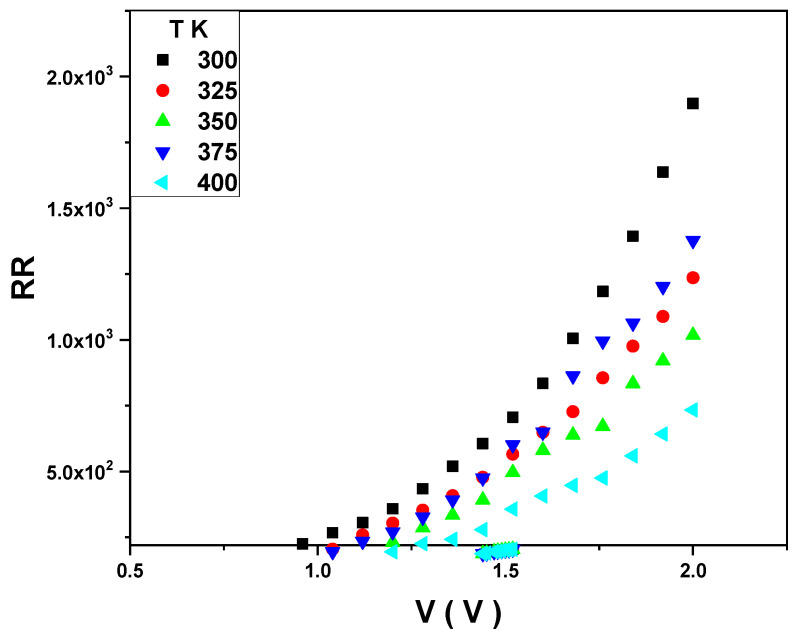
RR versus V at different temperatures for the Au/PVA/SiO_2_/p-Si/Al structure.

In addition to that, R_s_ and R_sh_ decrease with an increasing temperature. As illustrated in Figure 11, R_s_ and R_sh_ have a significant value due to the thin SiO_2_ layer between the PVA and Si layers. The variation of the junction resistance (Rj) with the applied voltage is shown in Figure 12. In contrast, the rectification ratio with a voltage at different temperatures is illustrated in Figure 13. The RR has good values at any temperature, confirming that the diode has a reasonable rectification.

## 3. Conclusions

In this study, we successfully fabricated an Au/PVA/SiO_2_/p-Si/Al structure, which was not previously reported. We investigated the variation of the dielectric constant (Є′), dielectric loss (Є″), and dielectric tangent (tanδ) across different frequencies, voltages, and temperatures. Our findings demonstrate an increase in the dielectric constant (Є′) and a reduction in both the dielectric loss (Є″) and tangent (tanδ). These results suggest that the Au/PVA/SiO_2_/p-Si/Al structure presents new opportunities for advancements in the semiconductor industry, particularly in capacitor manufacturing. Additionally, we systematically analyzed previous studies, examining the undesirable behaviors of Є′ and Є″. Cole–Cole diagrams of Є″ and Є′ under various voltages and temperatures were analyzed. We also performed I–V measurements and assessed electrical parameters such as the ideality factor, series resistance, shunt resistance, rectification ratio, and barrier height at different temperatures.

## 4. Materials and Methods

The Au/PVA/SiO_2_/p-Si/Al was synthesized by cleaning a single crystal wafer of silicon to eliminate all the contamination that exists on the surface. The silicon wafer was left to be oxidized in a furnace at 1170 k for thirty minutes, creating an oxide layer (SiO_2_). An aqueous gel solution of PVA was deposited on the surface of the SiO_2_/Si using the spin-coating technique. The Au/PVA/SiO_2_/p-Si/Al heterostructure was left to dry at a temperature of 223 k, and then a gold electrode was deposited on the top and aluminum as a lower electrode of the structure of the Au/PVA/SiO_2_/p-Si/Al using thermal evaporation.

## Figures and Tables

**Table 1:** Calculated ideality factor, series resistance, shunt resistance, and Au/PVA/SiO_2_/p-Si/Al barrier height at different temperatures.

T (k)	ɸ_b_ eV	R_sh_ Ω	R_s_ Ω	n
300	0.77	1.09 × 10^5^	3.38 × 10^3^	3.14
325	0.83	1.01 × 10^5^	2.44 × 10^3^	3.04
350	0.89	7.19 × 10^4^	2.01 × 10^3^	2.82
375	0.96	6.43 × 10^4^	1.80 × 10^3^	2.8
400	1.03	1.73 × 10^4^	1.31 × 10^3^	2.74
425	1.1	1.54 × 10^4^	1.40 × 10^3^	2.63

## Data Availability

The original contributions presented in the study are included in the article, further inquiries can be directed to the corresponding author.
